# Effects of Genetic Variants Previously Associated with Fasting Glucose and Insulin in the Diabetes Prevention Program

**DOI:** 10.1371/journal.pone.0044424

**Published:** 2012-09-11

**Authors:** Jose C. Florez, Kathleen A. Jablonski, Jarred B. McAteer, Paul W. Franks, Clinton C. Mason, Kieren Mather, Edward Horton, Ronald Goldberg, Dana Dabelea, Steven E. Kahn, Richard F. Arakaki, Alan R. Shuldiner, William C. Knowler, Diabetes Prevention Program Research Group

**Affiliations:** 1 Center for Human Genetic Research and Diabetes Research Center (Diabetes Unit), Massachusetts General Hospital, Boston, Massachusetts, United States of America; 2 Program in Medical and Population Genetics, Broad Institute, Cambridge, Massachusetts, United States of America; 3 Department of Medicine, Harvard Medical School, Boston, Massachusetts, United States of America; 4 The Biostatistics Center, George Washington University, Rockville, Maryland, United States of America; 5 Lund University Diabetes Center, Department of Clinical Sciences, Lund University, Malmö, Sweden; 6 Department of Nutrition, Harvard School of Public Health, Boston, Massachusetts, United States of America; 7 Diabetes Epidemiology and Clinical Research Section, National Institute of Diabetes and Digestive and Kidney Diseases, Phoenix, Arizona, United States of America; 8 Division of Endocrinology, Indiana University School of Medicine, Indianapolis, Indiana, United States of America; 9 Joslin Diabetes Center, Boston, Massachusetts, United States of America; 10 Lipid Disorders Clinic, Division of Endocrinology, Diabetes, and Metabolism, and the Diabetes Research Institute, Leonard M. Miller School of Medicine, University of Miami, Miami, Florida, United States of America; 11 Department of Preventive Medicine and Biometrics, University of Colorado at Denver and Health Sciences Center, Denver, Colorado, United States of America; 12 Division of Metabolism, Endocrinology and Nutrition, Department of Medicine, VA Puget Sound Health Care System and University of Washington, Seattle, Washington, United States of America; 13 Department of Medicine Clinical Research, University of Hawaii, Honolulu, Hawaii, United States of America; 14 Division of Endocrinology, Diabetes and Nutrition, Department of Medicine, University of Maryland School of Medicine, Baltimore, Maryland, United States of America; Universita Magna-Graecia di Catanzaro, Italy

## Abstract

Common genetic variants have been recently associated with fasting glucose and insulin levels in white populations. Whether these associations replicate in pre-diabetes is not known. We extended these findings to the Diabetes Prevention Program, a clinical trial in which participants at high risk for diabetes were randomized to placebo, lifestyle modification or metformin for diabetes prevention. We genotyped previously reported polymorphisms (or their proxies) in/near *G6PC2*, *MTNR1B*, *GCK*, *DGKB*, *GCKR*, *ADCY5*, *MADD*, *CRY2*, *ADRA2A*, *FADS1*, *PROX1*, *SLC2A2*, *GLIS3*, *C2CD4B*, *IGF1*, and *IRS1* in 3,548 Diabetes Prevention Program participants. We analyzed variants for association with baseline glycemic traits, incident diabetes and their interaction with response to metformin or lifestyle intervention. We replicated associations with fasting glucose at *MTNR1B* (*P*<0.001), *G6PC2* (*P* = 0.002) and *GCKR* (*P* = 0.001). We noted impaired β-cell function in carriers of glucose-raising alleles at *MTNR1B* (*P*<0.001), and an increase in the insulinogenic index for the glucose-raising allele at *G6PC2* (*P*<0.001). The association of *MTNR1B* with fasting glucose and impaired β-cell function persisted at 1 year despite adjustment for the baseline trait, indicating a sustained deleterious effect at this locus. We also replicated the association of *MADD* with fasting proinsulin levels (*P*<0.001). We detected no significant impact of these variants on diabetes incidence or interaction with preventive interventions. The association of several polymorphisms with quantitative glycemic traits is replicated in a cohort of high-risk persons. These variants do not have a detectable impact on diabetes incidence or response to metformin or lifestyle modification in the Diabetes Prevention Program.

## Introduction

Glucose homeostasis is tightly regulated. Control of its variation in non-diabetic individuals is influenced by familial factors, many of which are presumed to be heritable [1], [2]. In searching for genetic determinants of quantitative glycemic traits, candidate gene and genome-wide association studies (GWAS) conducted in populations of European descent have identified associations of fasting glucose with genetic variants in or near the genes that encode glucokinase (*GCK*; [3]), the glucose-6-phosphatase catalytic subunit (*G6PC2*; [4], [5]) and the melatonin receptor 1b (*MTNR1B*; [6], [7]). The Meta-Analysis of Glucose and Insulin-related traits Consortium (MAGIC) recently performed a global meta-analysis of 21 GWAS cohorts followed by replication in 26 studies, totaling >122,000 non-diabetic individuals for fasting glucose and >98,000 non-diabetic individuals for fasting insulin [8]. These efforts confirmed the *GCK*, *G6PC2* and *MTNR1B* associations, and uncovered associations of fasting glucose with single nucleotide polymorphisms (SNPs) in or near *DGKB*, *GCKR*, *ADCY5*, *MADD*, *CRY2*, *ADRA2A*, *FADS1*, *PROX1*, *SLC2A2*, *GLIS3*, *C2CD4B* and the type 2 diabetes genes *TCF7L2* and *SLC30A8*. In addition, SNPs in or near *IGF1*, *GCKR* and perhaps *IRS1* have been found to influence fasting insulin concentrations, a surrogate for insulin resistance. Of these loci, only *GCK*, *MTNR1B*, *DGKB*, *GCKR*, *ADCY5* and *PROX1* (besides *TCF7L2* and *SLC30A8*) were associated with type 2 diabetes at genome-wide significance levels, with several others (but not all) showing a consistent trend but not meeting the same stringent statistical threshold. This work has illustrated that genetic associations with quantitative intermediate traits may lead to the discovery of type 2 diabetes loci, but also that not all genetic loci that influence fasting glucose levels in healthy individuals necessarily contribute to type 2 diabetes pathogenesis.

The MAGIC investigators have also performed more detailed characterization of the mechanisms of glucose regulation influenced by these loci in white individuals [9]. In the Third National Health and Nutrition Examination Survey (NHANES III), a genetic risk score constructed with the glucose-raising alleles was shown to have consistent effects in other ethnic groups representative of the US population [10]. The Gene × Lifestyle interactions And Complex traits Involved in Elevated disease Risk (GLACIER) investigators showed that several of these loci associate with impaired fasting glucose (IFG) cross-sectionally and prospectively, and some have a progressively deleterious effect on fasting glucose [11]. Shortly thereafter, the Whitehall II investigators reported that a genetic risk score constructed with these variants was strongly associated with fasting glucose and remained stable over time [12]. Finally, we have recently shown that different genetic variants influence type 2 diabetes risk at distinct stages of the normoglycemia to IFG to type 2 diabetes progression, with *MTNR1B* and *GCK* exerting their effects preferentially in the normoglycemia to IFG transition [13].

To understand why some loci raise fasting glucose but do not increase type 2 diabetes risk, it is critical to establish whether their glucose-raising effects remain evident in the setting of impaired glucose tolerance (IGT), as glycemic context may modulate the strength of the genetic effect [13]. Furthermore, the impact of these loci on the prospective development of diabetes has not yet been reported. Finally, establishing whether and how distinct preventive interventions modulate these effects may facilitate the clinical translation of these findings and illuminate the specific genes and mechanisms by which these loci affect glycemic homeostasis. We concentrated on SNPs associated with fasting glucose, rather than those associated with 2-hour glucose [14], because 1) the two 2-hour glucose SNPs that are not already captured by fasting glucose-associated variants (*GIPR* and *VPS13C*) have no detectable impact on type 2 diabetes [15], 2) the ascertainment of DPP participants by the strict IGT definition is likely to bias the distribution of 2-hour glucose alleles, 3) longitudinal changes in 2-hour glucose among carriers of the 2-hour glucose-raising alleles have already been reported in a better suited population cohort [16], and 4) evidence obtained by the MAGIC investigators argues against an interaction of known 2-hour glucose loci with physical activity or body mass index (BMI) (Robert Scott, personal communication). We therefore genotyped the fasting glucose-associated SNPs in the multi-ethnic cohort of the Diabetes Prevention Program (DPP), and analyzed their relationships with glycemic measures at baseline and one year, the development of diabetes, and their potential interaction with preventive interventions on diabetes incidence.

**Table 1:** SNPs genotyped and their allele frequencies by ethnic group.

					Allele frequencies (%)				
SNP	Chromosome	Position(NCBI 36)	Nearest gene	Alleles(effect/other)	White(n = 1,617)	African-American(n = 592)	Hispanic(n = 475)	Asian(n = 125)	American Indian (n = 81)
**Fasting glucose**									
rs340874	1	184833918	*PROX1* *	C/T	55.9	19.8	41.2	42.3	35.4
rs573225	2	161653734	*G6PC2*	A/G	71.7	91.8	85.4	90.3	92.0
rs11708067	3	120438894	*ADCY5* *	A/G	79.4	85.9	77.3	91.2	70.0
rs11920090	3	168087406	*SLC2A2*	T/A	87.1	67.3	86.6	91.1	94.4
rs2191349	7	14947780	*DGKB* *	T/G	55.7	57.9	48.1	64.0	24.1
rs917793	7	44131132	*GCK* *	T/A	19.5	23.7	32.3	21.6	48.1
rs7034200	9	4244098	*GLIS3*	A/C	48.9	64.2	57.3	46.0	65.6
rs10885122	10	106670840	*ADRA2A*	G/T	88.0	35.6	84.3	86.8	88.9
rs11605924	11	45579933	*CRY2*	A/C	49.3	87.0	47.7	68.0	51.9
rs7944584	11	47035421	*MADD*	A/T	71.7	95.0	83.7	90.4	98.1
rs174550	11	57899714	*FADS1*	T/C	68.0	91.4	43.5	55.2	11.1
rs10830963	11	88799685	*MTNR1B* *	G/C	28.8	9.1	22.7	41.2	24.1
rs11071657	15	39256547	*C2CD4B*	A/G	64.4	86.9	53.7	70.0	37.0
**Fasting insulin**									
rs4675095	2	219495543	*IRS1*	A/T	93.3	98.5	84.8	85.6	69.1
rs855228	12	99957291	*IGF1*	T/C	84.3	40.9	76.1	65.4	79.0
**Fasting glucose and insulin**									
rs780094	2	27483120	*GCKR* *	C/T	59.6	81.7	62.2	66.8	88.9

* Loci previously associated with type 2 diabetes at genome-wide levels of statistical significance. The allele previously associated with higher levels of the trait (effect allele) is shown first; allele frequencies correspond to the effect allele. Gene names: *PROX1*, prospero homeobox 1; *G6PC2*, glucose-6-phosphatase, catalytic, 2; *ADCY5*, adenylate cyclase 5; *SLC2A2*, solute carrier family 2, member 2; *DGKB*, diacylglycerol kinase, beta 90 kDa; *GCK*, glucokinase; *GLIS3*, GLIS family zinc finger 3; *ADRA2A*, adrenergic, alpha-2A-, receptor; *CRY2*, cryptochrome 2; *MADD*, MAP-kinase activating death domain; *FADS1*, fatty acid desaturase 1; *MTNR1B*, melatonin receptor 1B; *C2CD4B*, C2 calcium-dependent domain containing 4B; *IRS1*, insulin receptor substrate 1; *IGF1*, insulin-like growth factor 1; *GCKR*, glucokinase regulator.

## Methods

### The Diabetes Prevention Program

The DPP study design and baseline characteristics of the participants have been described previously [17], [18]. Briefly, the DPP was designed to test whether intensive lifestyle modification or pharmacologic interventions with metformin or troglitazone prevent or delay the onset of diabetes in individuals at high risk. The trial, conducted from 1996 to 2001 in 27 US-based medical centers, included 3,234 participants randomized to intensive lifestyle modification (goal >7% weight loss and >150 min/week of physical activity), metformin (850 mg twice daily), or placebo; the fourth arm, comprising 585 additional participants randomized to troglitazone, was terminated early because of concerns with hepatotoxicity. For enrollment, participants had to have a fasting glucose between 95–125 mg/dL and IGT (2h-glucose between 140–199 mg/dL after a 75-gram oral glucose tolerance test [OGTT]). Of the total 3,819 DPP participants, 3,548 had DNA and consented to genetic investigation: 56.4% were of European descent, 20.2% African American, 16.8% Hispanic, 4.3% Asian and 2.4% American Indian by self-report. Their mean age was 51 years and mean BMI was 34.0 kg/m^2^. The primary endpoint (diabetes incidence, ascertained biannually and confirmed on a second occasion) was reached in nearly 38% of participants randomized to the placebo arm after a mean of 3.2 years of follow-up; there was a 58% reduction of diabetes incidence in the lifestyle intervention group and a 31% reduction in the metformin group compared to placebo [19]. For the purposes of this study, participants randomized to troglitazone were excluded, leaving a total of 2,890 individuals with valid genotypes for analysis. Institutional Review Board approval was obtained by each participating site, and all participants included in this report provided written informed consent for the main study and for subsequent genetic investigations.

**Table 2:** Nominal genotypic associations with quantitative traits at baseline.

SNP	Nearest gene	Alleles (effect/other)	Trait	LS Means (95% CI)	Additive *P* value	Pairwise *P* values
rs573225	*G6PC2*	A/G	FG (mg/dL)	AA 106.7 (106.2–107.2)	0.002	AA vs AG 0.002
				AG 105.6 (104.9–106.3)		AA vs GG 0.31
				GG 105.8 (104.5–107.1)		AG vs GG 0.73
			Fins (µU/mL)	AA 24.44 (23.65–25.26)	0.006	AA vs AG 0.31
				AG 24.97 (23.87–26.11)		AA vs GG 0.005
				GG 27.71 (25.56–30.05)		AG vs GG 0.02
			Ins Index	AA 1.25 (1.20–1.31)	0.002	AA vs AG 0.16
				AG 1.20 (1.13–1.28)		AA vs GG 0.003
				GG 1.04 (0.92–1.17)		AG vs GG 0.03
			ISI	AA 0.155 (0.15–0.161)	0.03	AA vs AG 0.62
				AG 0.154 (0.147–0.161)		AA vs GG 0.01
				GG 0.138 (0.127–0.15)		AG vs GG 0.03
			DIo	AA 0.049 (0.047–0.051)	<0.001	AA vs AG 0.03
				AG 0.046 (0.043–0.049)		AA vs GG <0.001
				GG 0.037 (0.033–0.042)		AG vs GG <0.001
rs11708067	*ADCY5*	A/G	Fins (µU/mL)	AA 24.05 (23.24–24.88)	0.001	AA vs AG 0.001
				AG 25.85 (24.79–26.95)		AA vs GG 0.53
				GG 25.29 (23.12–27.67)		AG vs GG 0.64
			ISI	AA 0.158 (0.153–0.164)	0.004	AA vs AG 0.003
				AG 0.148 (0.141–0.154)		AA vs GG 0.72
				GG 0.151 (0.138–0.166)		AG vs GG 0.72
rs11920090	*SLC2A2*	T/A	DIo	AA 0.042 (0.037–0.049)	0.006	AA vs AT 0.27
				AT 0.046 (0.043–0.049)		AA vs TT 0.04
				TT 0.049 (0.047–0.051)		AT vs TT 0.03
rs7944584	*MADD*	A/T	Proins (pmol/L)	AA 16.4 (15.9–16.92)	<0.001	AA vs AT <0.001
				AT 14.98 (14.34–15.65)		AA vs TT <0.001
				TT 13.53 (12.46–14.68)		AT vs TT 0.01
rs174550	*FADS1*	T/C	Fins (µU/mL)	TT 23.78 (22.83–24.78)	0.008	TT vs CT 0.06
				CT 24.97 (23.96–26.03)		TT vs CC 0.06
				CC 25.52 (24.25–26.86)		CT vs CC 0.47
			ISI	CC 0.149 (0.141–0.157)	0.01	TT vs CT 0.09
				CT 0.153 (0.146–0.159)		TT vs CC 0.09
				TT 0.160 (0.153–0.167)		CT vs CC 0.46
rs10830963	*MTNR1B*	G/C	FG (mg/dL)	GG 108.7 (107.6–109.9)	<0.001	GG vs CG 0.02
				CG 107.3 (106.7–108.0)		GG vs CC <0.001
				CC 105.6 (105.1–106.2)		CG vs CC <0.001
			Proins (pmol/L)	GG 15.88 (14.80–17.03)	0.009	GG vs CG 0.66
				CG 15.43 (14.85–16.04)		GG vs CC 0.66
				CC 16.44 (15.90–17.00)		CG vs CC 0.003
			Ins Index	GG 1.17 (1.05–1.29)	0.01	GG vs CG 0.74
				CG 1.19 (1.12–1.25)		GG vs CC 0.21
				CC 1.27 (1.21–1.33)		CG vs CC 0.05
rs855228	*IGF1*	T/C	FG (mg/dL)	TT 106.1 (105.5–106.7)	0.01	TT vs CT 0.37
				CT 106.4 (105.8–107.0)		TT vs CC 0.02
				CC 107.7 (106.6–108.7)		CT vs CC 0.43
rs780094	*GCKR*	C/T	FG (mg/dL)	CC 106.8 (106.2–107.3)	0.001	CC vs CT 0.12
				CT 106.3 (105.6–106.9)		CC vs TT 0.003
				TT 105.2 (104.3–106.1)		CT vs TT 0.04

FG, fasting glucose; Fins, fasting insulin; Ins Index, insulinogenic index; ISI, insulin sensitivity index; DIo, oral disposition index; Proins, fasting proinsulin adjusted for fasting insulin. To convert glucose mg/dL to mmol/L, divide by 18.01. To convert insulin µU/ml to pmol/L to, multiply by 6.0.

### Quantitative Glycemic Traits

We calculated the insulin sensitivity index (ISI) as 22.5/[(fasting insulin × fasting glucose)/18.01]; the ISI is the reciprocal of insulin resistance calculated by homeostasis model assessment (HOMA-IR) [20]. We estimated insulin secretion by the insulinogenic index using the formula [(insulin at 30 min)-(insulin at 0 min)]/[(glucose at 30 min)-(glucose at 0 min)]. The oral disposition index (DIo) was calculated as 1/fasting insulin × insulinogenic index [21]. We studied genetic associations with these measures at baseline and at 1 year: we chose one year because changes in weight were most pronounced at that time point, and it contained the highest number of participants with available measures.

**Table 3:** Associations with quantitative traits at one year.

			FG		Fins		Proins		Ins Index		ISI		DIo	
SNP	Nearest gene	Alleles(effect/other)	*P* int	*P* assoc	*P* int	*P* assoc	*P* int	*P* assoc	*P* int	*P* assoc	*P* int	*P* assoc	*P* int	*P* assoc
rs340874	*PROX1*	C/T	0.81	0.47	0.90	0.15	0.99	0.08	0.42	0.99	0.89	0.14	0.63	0.22
rs573225	*G6PC2*	A/G	0.96	0.17	0.08	0.88	0.34	0.91	0.77	0.20	0.11	0.66	0.81	0.08
rs11708067	*ADCY5*	A/G	0.80	0.27	0.22	0.98	0.31	0.85	0.46	0.32	0.20	0.80	0.86	0.52
rs11920090	*SLC2A2*	T/A	0.88	0.49	0.24	0.53	0.79	0.38	0.59	0.98	0.25	0.49	0.69	0.89
rs2191349	*DGKB*	T/G	0.79	0.41	0.04	–	0.09	0.80	0.50	0.55	0.07	0.84	0.84	0.99
rs917793	*GCK*	T/A	0.07	0.12	0.39	0.12	0.08	0.31	0.86	0.23	0.24	0.08	0.42	0.07
rs7034200	*GLIS3*	A/C	0.72	0.98	0.02	–	0.25	0.56	0.94	0.94	0.03	–	0.13	0.64
rs10885122	*ADRA2A*	G/T	0.82	0.14	0.20	0.60	0.16	0.46	0.29	0.18	0.25	0.44	0.31	0.03
rs11605924	*CRY2*	A/C	0.20	0.56	0.13	0.46	0.76	0.80	0.40	0.28	0.13	0.40	0.21	0.58
rs7944584	*MADD*	A/T	0.04	–	0.73	0.80	0.63	0.30	0.11	0.10	0.63	0.83	0.36	0.29
rs174550	*FADS1*	T/C	0.58	0.20	0.87	0.09	0.73	0.23	0.76	0.97	0.86	0.07	0.64	0.65
rs10830963	*MTNR1B*	G/C	0.68	0.003	0.17	0.37	0.27	0.41	0.69	0.002	0.16	0.90	0.96	0.08
rs11071657	*C2CD4B*	A/G	0.04	–	0.96	0.42	0.55	0.97	0.38	0.09	0.98	0.54	0.35	0.048
rs4675095	*IRS1*	A/T	0.21	0.99	0.67	0.26	0.68	0.59	0.53	0.55	0.71	0.30	0.62	0.62
rs855228	*IGF1*	T/C	0.39	0.60	0.15	0.27	0.52	0.87	0.36	0.72	0.15	0.24	0.63	0.75
rs780094	*GCKR*	C/T	0.57	0.76	0.07	0.22	0.25	0.38	0.17	0.92	0.09	0.26	0.43	0.28

FG, fasting glucose; Fins, fasting insulin; Ins Index, insulinogenic index; ISI, insulin sensitivity index; DIo, oral disposition index; Proins, fasting proinsulin adjusted for fasting insulin. *P* int denotes the *P* value for the genotype × intervention interaction test; *P* assoc denotes the *P* value for the main effect association in the full cohort when *P* int >0.05.

**Table 4:** Levels of quantitative glycemic traits at one year by genotype and treatment arm at loci with a nominally significant interaction.

			Placebo		Metformin		Lifestyle	
SNP gene	Alleles (effect/other)	Trait	LS Means (95% CI)	*P* values	LS Means (95% CI)	*P* values	LS Means (95% CI)	*P* values
rs2191349	T/G	Fins	GG 24.71 (22.96–26.59)	GG/GT: 0.99	GG 22.62 (21.02–24.34)	GG/GT: 0.21	GG 18.43 (17.03–19.94)	GG/GT: 0.99
*DGKB*		(µU/mL)	GT 24.99 (23.59–26.48)	GG/TT: 0.99	GT 21.30 (20.06–22.62)	GG/TT: 0.04	GT 19.01 (17.82–20.28)	GG/TT: 0.99
			TT 25.71 (24.00–27.54)	GT/TT: 0.99	TT 20.41 (19.03–21.88)	GT/TT: 0.21	TT 19.05 (17.71–20.51)	GT/TT: 0.99
rs7034200	A/C	Fins	AA 24.86 (23.27–26.55)	AA/AC: 0.99	AA 22.54 (21.06–24.12)	AA/AC: 0.12	AA 18.01 (16.76–19.36)	AA/AC: 0.28
*GLIS3*		(µU/mL)	AC 25.23 (23.83–26.73)	AA/CC: 0.99	AC 21.14 (19.87–22.50)	AA/CC: 0.05	AC 19.20 (18.02–20.45)	AA/CC: 0.28
			CC 25.07 (23.18–27.12)	AC/CC: 0.99	CC 20.45 (19.00–22.01	AC/CC: 0.37	CC 19.47 (17.96–21.11)	AC/CC: 0.74
		ISI	AA 0.153 (0.142–0.164)	AA/AC: 0.99	AA 0.175 (0.163–0.189)	AA/AC: 0.14	AA 0.222 (0.205–0.240)	AA/AC: 0.28
			AC 0.152 (0.143–0.162)	AA/CC: 0.99	AC 0.187 (0.175–0.200)	AA/CC: 0.06	AC 0.207 (0.193–0.221)	AA/CC: 0.28
			CC 0.151 (0.139–0.165)	AC/CC: 0.99	CC 0.194 (0.179–0.210)	AC/CC: 0.36	CC 0.204 (0.186–0.222)	AC/CC: 0.74
rs7944584	A/T	FG	AA 106.8 (105.5–108.1)	AA/AT: 0.008	AA 102.4 (101.3–103.5)	AA/AT: 0.99	AA 102.1 (101.0–103.2)	AA/AT: 0.99
*MADD*		(mg/dL)	AT 104.3 (102.6–106.1)	AA/TT: 0.50	AT 102.7 (101.1–104.2)	AA/TT: 0.99	AT 101.5 (99.98–103.1)	AA/TT: 0.99
			TT 104.8 (101.4–108.3)	AT/TT: 0.78	TT 101.3 (98.45–104.3)	AT/TT: 0.99	TT 101.4 (98.65–104.2)	AT/TT: 0.99
rs11071657	A/G	FG	AA 107.1 (105.6–108.7)	AA/AG: 0.41	AA 102.3 (101.0–103.6)	AA/AG: 0.99	AA 101.9 (100.6–103.2)	AA/AG: 0.96
*C2CD4B*		(mg/dL)	AG 105.9 (104.5–107.4)	AA/GG: 0.41	AG 102.7 (101.4–104.0)	AA/GG: 0.99	AG 101.7 (100.4–103.0)	AA/GG: 0.96
			GG 105.3 (103.1–107.6)	AG/GG: 0.63	GG 102.0 (100.2–103.9)	AG/GG: 0.99	GG 102.7 (100.8–104.7)	AG/GG: 0.96

*P* values for pairwise comparisons between genotypic groups are shown, with groups separated by a “/”. Fins, fasting insulin (µU/mL); ISI, insulin sensitivity index; FG, fasting glucose (mg/dL). To convert glucose mg/dL to mmol/L, divide by 18.01. To convert insulin µU/ml to pmol/L to, multiply by 6.0.

### SNP Selection and Genotyping

We genotyped the index SNPs associated with fasting glycemic traits reported by the MAGIC investigators [8]. Where assay design failed we selected proxies based on linkage disequilibrium in the HapMap CEU population: rs573225 for rs560887 in *G6PC2,* r^2^ = 0.961; rs917793 for rs4607517 in *GCK,* r^2^ = 1.0; and rs855228 for rs35767 in *IGF1*, r^2^ = 0.915. DNA was extracted from peripheral blood leukocytes and quantitated as previously described [22]. Genotyping was carried out by allele-specific primer extension of multiplex amplified products and detection using matrix-assisted laser desorption ionization time-of-flight mass spectrometry on a Sequenom iPLEX platform [23]. Genotyping success rate was ≥98.5%. Because results for the two previously known type 2 diabetes genes *TCF7L2* and *SLC30A8* have been reported elsewhere [22], [24], [25], they are not presented here.

**Figure 1:**
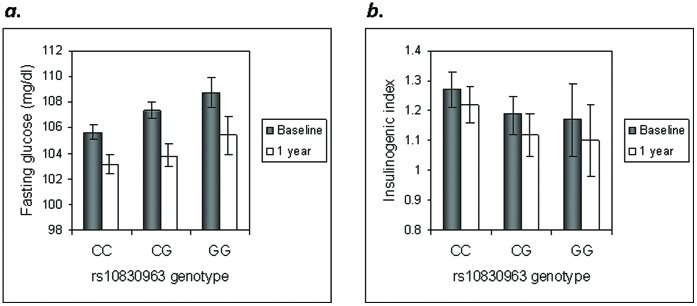
Effect of genotype at *MTNR1B* rs10830963 on glycemic traits at baseline and one year. Fasting glucose is shown in panel (a) and the insulinogenic index is shown in panel (b). Because no significant SNP × intervention interaction was found, the full cohort was analyzed in aggregate. Fasting glucose is higher (*P* = 0.003) and the insulinogenic index is lower (*P* = 0.002) in carriers of the G risk allele after one year, even after adjustment for the corresponding baseline levels. Least-square means (±95% CI) are shown. To convert glucose mg/dL to mmol/L, divide by 18.01.

### Statistical Analyses

We used Cox proportional hazards regression models with genotype, intervention and their interactions as the independent variables predicting time to diabetes over mean 3.2 years follow-up. We adjusted for gender, age at enrollment, ethnicity, treatment arm, and baseline BMI. For the quantitative glycemic traits, we employed generalized mixed models to test additive effects of genotype on baseline log-transformed quantitative traits, and on the same traits after one year of intervention adjusted for the baseline value, age, sex, self-reported ethnicity, BMI and treatment arm. We note that these SNPs have been associated with glycemic traits at genome-wide levels of significance, and therefore their prior probability of true effects is many orders of magnitude higher than the genome average. As our analyses represent further characterization of each of these established loci, we selected a *P* value threshold of 0.05. Finally, we also tested for any evidence of epistatic interactions between the *MTNR1B* SNP rs10830963 and the *G6PC2* SNP rs573225, both of which have significant effects on fasting glucose in the DPP, by including appropriate interaction terms at baseline and one year.

**Table 5:** Diabetes incidence by genotype at each locus, in the overall cohort and stratified by treatment arm.

SNP	Nearest gene	Alleles	SNP * Tx	Treatment adjusted HR(95% CI)	*P*-value	PLACEBO HR(95% CI)	*P*-value	METFORMIN HR(95% CI)	*P*-value	LIFESTYLE HR(95% CI)	*P*-value
rs340874	*PROX1* *	C (vs T)	N	0.88 (0.78–0.98)	0.02	0.85 (0.71–1.01)	0.06	0.92 (0.75–1.12)	0.39	0.86 (0.68–1.08)	0.20
rs573225	*G6PC2*	A (vs G)	N	1.11 (0.97–1.27)	0.14	0.96 (0.77–1.19)	0.70	1.18 (0.94–1.47)	0.15	1.27 (0.98–1.64)	0.07
rs11708067	*ADCY5* *	A (vs G)	N	1.06 (0.92–1.23)	0.38	1.04 (0.84–1.28)	0.73	1.08 (0.84–1.35)	0.60	1.10 (0.82–1.47)	0.51
rs11920090	*SLC2A2*	T (vs A)	N	1.02 (0.88–1.19)	0.75	1.08 (0.86–1.33)	0.56	1.00 (0.77–1.30)	0.99	0.99 (0.71–1.37)	0.93
rs2191349	*DGKB* *	T (vs G)	N	1.06 (0.94–1.18)	0.34	1.05 (0.88–1.27)	0.56	1.10 (0.90–1.33)	0.34	1.01 (0.80–1.27)	0.96
rs917793	*GCK* *	T (vs A)	N	0.96 (0.84–1.10)	0.59	0.87 (0.70–1.07)	0.20	1.14 (0.90–1.44)	0.29	0.92 (0.69–1.22)	0.56
rs7034200	*GLIS3*	A (vs C)	N	1.00 (0.89–1.12)	1.00	0.90 (0.75–1.08)	0.22	1.04 (0.85–1.27)	0.68	1.15 (0.91–1.47)	0.25
rs10885122	*ADRA2A*	G (vs T)	N	1.03 (0.91–1.16)	0.63	1.01 (0.84–1.22)	0.89	1.03 (0.84–1.28)	0.76	1.08 (0.84–1.39)	0.56
rs11605924	*CRY2*	A (vs C)	N	1.01 (0.90–1.12)	0.91	0.93 (0.78–1.10)	0.40	1.06 (0.88–1.28)	0.56	1.09 (0.87–1.37)	0.47
rs7944584	*MADD*	A (vs T)	N	0.93 (0.80–1.08)	0.29	0.86 (0.69–1.08)	0.20	0.89 (0.69–1.14)	0.35	1.11 (0.84–1.47)	0.47
rs174550	*FADS1*	T (vs C)	N	0.94 (0.83–1.05)	0.26	0.95 (0.80–1.14)	0.57	0.98 (0.80–1.19)	0.81	0.86 (0.67–1.09)	0.21
rs10830963	*MTNR1B* *	G (vs C)	N	1.07 (0.94–1.22)	0.29	1.20 (0.98–1.47)	0.07	1.01 (0.80–1.26)	0.95	0.95 (0.73–1.24)	0.69
rs11071657	*C2CD4B*	A (vs G)	N	0.93 (0.83–1.05)	0.26	0.93 (0.77–1.12)	0.43	0.92 (0.75–1.14)	0.42	0.96 (0.75–1.22)	0.72
rs4675095	*IRS1*	A (vs T)	N	0.96 (0.78–1.18)	0.68	0.93 (0.67–1.30)	0.69	1.16 (0.84–1.59)	0.37	0.71 (0.44–1.15)	0.17
rs855228	*IGF1*	T (vs C)	N	1.09 (0.97–1.23)	0.14	1.04 (0.87–1.25)	0.66	1.16 (0.95–1.43)	0.15	1.12 (0.88–1.43)	0.38
rs780094	*GCKR* *	C (vs T)	N	0.96 (0.85–1.08)	0.48	0.91 (0.75–1.10)	0.33	1.01 (0.82–1.23)	0.93	0.96 (0.75–1.22)	0.72

* Loci previously associated with type 2 diabetes. Effect allele denotes the allele associated with higher glucose or insulin levels in MAGIC. There are no significant SNP × treatment interactions. One nominally significant *P* value for association with diabetes incidence is not consistent with the expected direction of effect.

## Results

### Baseline Associations

The SNPs genotyped, their chromosomal location, the nearest gene and their allele frequencies in the five DPP ethnic groups are shown in Table 1. Allele frequencies were comparable to those previously reported by MAGIC in Europeans [8] and NHANES III in non-Hispanic whites, African Americans and US Hispanics [10].

We tested associations of these SNPs with baseline fasting glucose, fasting insulin, fasting proinsulin adjusted for fasting insulin, the insulinogenic index, the ISI and the DIo in this multiethnic cohort of individuals with IGT. We replicated associations with fasting glucose at *G6PC2* (*P* = 0.002), *MTNR1B* (*P*<0.001) and *GCKR* (*P* = 0.001). We also replicated associations of the glucose-raising allele with *reduced* insulinogenic index at *MTNR1B* and *increased* insulinogenic and disposition indices at *G6PC2*. We again noted a strong association of *MADD* with fasting proinsulin levels, adjusted for concomitant insulin (*P*<0.001). All nominally significant (*P*<0.05) associations and corresponding trait distributions are shown in Table 2.

### Associations at One Year

We tested whether the metformin or lifestyle preventive interventions interacted with each SNP to modulate quantitative glycemic traits at one year. We adjusted one-year traits for the corresponding baseline trait, to indicate change in each variable during active treatment. Where no nominally significant interaction with treatment was found, SNP main effects on the one-year trait were tested in the whole cohort with an adjustment for treatment arm; if an interaction was detected at *P*<0.05, analyses were stratified by treatment arm (Table 3). Nominally significant interactions were found for *DGKB* and fasting insulin, *GLIS3* and both fasting insulin and ISI, and both *MADD* and *C2CD4B* and fasting glucose. Least-square means for each genotype group and the corresponding pairwise comparisons are shown in Table 4.

At *MTNR1B*, the glucose-raising allele continued to have a significant main effect on raising fasting glucose and lowering the insulinogenic index at one year (Figure 1). Because one-year traits are adjusted for the baseline level, this effect is indicative of a worsening deleterious effect of this locus on β-cell function. We further explored the concordant effects of SNPs at *MTNR1B* and *G6PC2* on fasting glucose but discordant effects for insulinogenic index by testing for epistatic interactions between the two on fasting glucose at baseline and one year: the interaction terms were not statistically significant.

### Diabetes Incidence

We tested whether the metformin or lifestyle preventive interventions interact with each SNP on the risk of developing diabetes during 3.2 years of mean follow-up. As no nominal interactions were found, the effects of each SNP on diabetes incidence were evaluated in the full cohort while adjusting for treatment arm; stratified analyses are also shown (Table 5). The only nominal association with diabetes incidence was found for the glucose-lowering allele at *PROX1* (*P* = 0.02), in a direction opposite to that reported in case-control analyses in MAGIC, where the C allele increased type 2 diabetes risk (odds ratio 1.07 [95% CI 1.05–1.09], *P* = 7.2×10^−10^) [8].

## Discussion

The MAGIC investigators reported a number of loci that influence fasting glucose and fasting insulin levels in non-diabetic populations of European descent; only a few of the loci were also associated with type 2 diabetes at genome-wide levels of significance [8]. The authors speculated that it is not the mere elevation in fasting glucose, but how fasting glucose is raised, that determines overall β-cell dysfunction and future type 2 diabetes risk. However, whether these loci exert their action on fasting glucose in the initial stages of diabetes progression (e.g. from normoglycemia to impaired glucose regulation) or later (e.g. from IGT to type 2 diabetes) is not known. In the GLACIER cohort, eleven loci (including the known type 2 diabetes genes *TCF7L2* and *SLC30A8*) were nominally associated with IFG cross-sectionally, and *MTNR1B* and *G6PC2* were also associated with development of IFG in longitudinal analyses [11]. We have recently shown that among type 2 diabetes-associated loci, risk alleles at *MTNR1B*, *GCK* and *SLC30A8* confer a stronger rate of progression from normoglycemia to IFG than from IFG to type 2 diabetes [13]. Here we extend these findings by testing these SNPs from the IGT to type 2 diabetes transition, and by assessing their effects on quantitative glycemic traits at baseline and one year in a multiethnic cohort of persons with IGT.

We have demonstrated that the three loci with the strongest reported effect on fasting glucose (*MTNR1B*, *GCKR* and *G6PC2*) have consistent effects in the DPP. All three were known to be associated with fasting glucose prior to the MAGIC GWAS meta-analysis [4], [5], [6], [7], [26], [27], [28]. Power may have been limiting to detect the other reported associations [24].

We have also confirmed that the glucose-raising allele at *MTNR1B* is associated with a reduced insulinogenic index, as measured during the initial phase of insulin secretion during an OGTT [9], [29]. As shown by Lyssenko and coworkers, the deleterious effects of this allele on β-cell function persist over time; while they noted such worsening over 24 years of follow-up [29], here we see such effects over a much shorter time span (one year). In GLACIER a similar non-significant trend was noted over 10 years of follow-up [11], although a consistent effect was not detected in the Whitehall II study [12]. Because *MTNR1B* does increase risk of type 2 diabetes [8], this pattern of sustained deterioration suggests that identifying these individuals early in their glycemic progression may be beneficial in prevention efforts.

In contrast, the glucose-raising allele at *G6PC2* is associated with superior β-cell function on dynamic testing; this has been shown previously [9], [30], and is consistent with the role of this gene product in regulating hepatic glucokinase and its null effect on type 2 diabetes risk [8]. We found no evidence in support of a non-additive interaction between *MTNR1B* and *G6PC2* on fasting glucose at baseline or one year. The strong effect of the *MADD* locus on fasting proinsulin levels is also confirmed [9], [31]; because this association is adjusted for concomitant insulin levels, it reflects an increased secretion of insulin precursors out of proportion to the degree of basal insulin resistance. The other nominal associations newly reported here do not withstand correction for the multiple statistical tests performed, and should be considered hypothesis-generating requiring confirmation in independent studies.

In summary, the strongest effects of genetic loci on fasting glucose in non-diabetic individuals of European descent are also evident in a multiethnic cohort with IGT. The deleterious influence of the glucose-raising allele at *MTNR1B* on β-cell function appears to worsen with time, and this effect is evident in as short a time as one year. Genetic testing may identify a subset of patients with IGT more likely to respond to preventive interventions [32].

## Supporting Information

Appendix S1 **DPP Research Group.**
(DOC)
