# Supplementary material for: Effects of Genetic Variants Previously Associated with Fasting Glucose and Insulin in the Diabetes Prevention Program
Source: PLoS One. 2012 Sep 11;7(9):e44424. doi: 10.1371/journal.pone.0044424 (PMC3439414; doi:10.1371/journal.pone.0044424)
Supplement: Appendix S1 — DPP Research Group. (DOC) [file pone.0044424.s001.doc]

Pennington Biomedical Research Center (Baton Rouge, LA)

George A. Bray, MD*

Iris W. Culbert, BSN, RN, CCRC**

Catherine M. Champagne, PhD, RD

Barbara Eberhardt, RD, LDN

Frank Greenway, MD

Fonda G. Guillory, LPN

April A. Herbert, RD

Michael L. Jeffirs, LPN

Betty M. Kennedy, MPA

Jennifer C. Lovejoy, PhD

Laura H. Morris, BS

Lee E. Melancon, BA, BS

Donna Ryan, MD

Deborah A. Sanford, LPN

Kenneth G. Smith, BS, MT

Lisa L. Smith, BS

Julia A. St.Amant, RTR

Richard T. Tulley, PhD

Paula C. Vicknair, MS, RD

Donald Williamson, PhD

Jeffery J. Zachwieja, PhD

# University of Chicago (Chicago, IL)

Kenneth S. Polonsky, MD*

Janet Tobian, MD, PhD*

David Ehrmann, MD*

Margaret J. Matulik, RN, BSN**

Bart Clark, MD

Kirsten Czech, MS

Catherine DeSandre, BA

Ruthanne Hilbrich, RD

Wylie McNabb, EdD

Ann R. Semenske, MS, RD

***Jefferson Medical College* (Philadelphia, PA)**

Jose F. Caro, MD*

Pamela G. Watson, RN, ScD*

Barry J. Goldstein, MD, PhD*

Kellie A. Smith, RN, MSN**

Jewel Mendoza, RN, BSN**

Renee Liberoni, MPH

Constance Pepe, MS, RD

John Spandorfer, MD

***University of Miami* (Miami, FL)**

Richard P. Donahue, PhD*

Ronald B. Goldberg, MD*

Ronald Prineas, MD, PhD*

Patricia Rowe, MPA**

Jeanette Calles, MSEd

Paul Cassanova-Romero, MD

Hermes J. Florez, MD

Anna Giannella, RD, MS

Lascelles Kirby, MS

Carmen Larreal

Valerie McLymont, RN

Jadell Mendez

Juliet Ojito, RN

Arlette Perry, PhD

Patrice Saab, PhD

***The University of Texas Health Science Center* (San Antonio, TX)**

Steven M. Haffner, MD, MPH*

Maria G. Montez, RN, MSHP, CDE**

Carlos Lorenzo, MD, PhD

Arlene Martinez, RN, BSN, CDE

***University of Colorado* (Denver, CO)**

Richard F. Hamman, MD, DrPH*

Patricia V. Nash, MS**

Lisa Testaverde, MS**

Denise R. Anderson, RN, BSN

Larry B. Ballonoff, MD

Alexis Bouffard, MA,

B. Ned Calonge, MD, MPH

Lynne Delve

Martha Farago, RN

James O. Hill, PhD

Shelley R. Hoyer, BS

Bonnie T. Jortberg, MS, RD, CDE

Dione Lenz, RN, BSN

Marsha Miller, MS, RD

David W. Price, MD

Judith G. Regensteiner, PhD

Helen Seagle, MS, RD

Carissa M. Smith, BS

Sheila C. Steinke, MS

Brent VanDorsten, PhD

***Joslin Diabetes Center* (Boston, MA)**

Edward S. Horton, MD*

Kathleen E. Lawton, RN**

Ronald A. Arky, MD

Marybeth Bryant

Jacqueline P. Burke, BSN

Enrique Caballero, MD

Karen M. Callaphan, BA

Om P. Ganda, MD

Therese Franklin

Sharon D. Jackson, MS, RD, CDE

Alan M. Jacobsen, MD

Lyn M. Kula, RD

Margaret Kocal, RN, CDE

Maureen A. Malloy, BS

Maryanne Nicosia, MS, RD

Cathryn F. Oldmixon, RN

Jocelyn Pan, BS, MPH

Marizel Quitingon

Stacy Rubtchinsky, BS

Ellen W. Seely, MD

Dana Schweizer, BSN

Donald Simonson, MD

Fannie Smith, MD

Caren G. Solomon, MD, MPH

James Warram, MD

***VA Puget Sound Health Care System and University of Washington* (Seattle, WA)**

Steven E. Kahn, MB, ChB*

Brenda K. Montgomery, RN, BSN, CDE**

Wilfred Fujimoto, MD

Robert H. Knopp, MD

Edward W. Lipkin, MD

Michelle Marr, BA

Dace Trence, MD

***University of Tennessee* (Memphis, TN)**

Abbas E. Kitabchi, PhD, MD, FACP*

Mary E. Murphy, RN, MS, CDE, MBA**

William B. Applegate, MD, MPH

Michael Bryer-Ash, MD

Sandra L. Frieson, RN

Raed Imseis, MD

Helen Lambeth, RN, BSN

Lynne C. Lichtermann, RN, BSN

Hooman Oktaei, MD

Lily M.K. Rutledge, RN, BSN

Amy R. Sherman, RD, LD

Clara M. Smith, RD, MHP, LDN

Judith E. Soberman, MD

Beverly Williams-Cleaves, MD

***Northwestern University’s Feinberg School of Medicine* (Chicago, IL)**

Boyd E. Metzger, MD*

Mariana K. Johnson, MS, RN**

Catherine Behrends

Michelle Cook, MS

Marian Fitzgibbon, PhD

Mimi M. Giles, MS, RD

Deloris Heard, MA

Cheryl K.H. Johnson, MS, RN

Diane Larsen, BS

Anne Lowe, BS

Megan Lyman, BS

David McPherson, MD

Mark E. Molitch, MD

Thomas Pitts, MD

Renee Reinhart, RN, MS

Susan Roston, RN, RD

Pamela A. Schinleber, RN, MS

***Massachusetts General Hospital* (Boston, MA)**

David M. Nathan, MD*

Charles McKitrick, BSN**

Heather Turgeon, BSN**

Kathy Abbott

Ellen Anderson, MS, RD

Laurie Bissett, MS, RD

Enrico Cagliero, MD

Jose C. Florez, MD, PhD^+^

Linda Delahanty, MS, RD

Valerie Goldman, MS, RD

Alexandra Poulos

***University of California-San Diego* (San Diego, CA)**

Jerrold M. Olefsky, MD*

Mary Lou Carrion-Petersen, RN, BSN**

Elizabeth Barrett-Connor, MD

Steven V. Edelman, MD

Robert R. Henry, MD

Javiva Horne, RD

Simona Szerdi Janesch, BA

Diana Leos, RN, BSN

Sundar Mudaliar, MD

William Polonsky, PhD

Jean Smith, RN

Karen Vejvoda, RN, BSN, CDE, CCRC

***St. Luke’s-Roosevelt Hospital* (New York, NY)**

F. Xavier Pi-Sunyer, MD*

Jane E. Lee, MS**

David B. Allison, PhD

Nancy J. Aronoff, MS, RD

Jill P. Crandall, MD

Sandra T. Foo, MD

Carmen Pal, MD

Kathy Parkes, RN

Mary Beth Pena, RN

Ellen S. Rooney, BA

Gretchen E.H. Van Wye, MA

Kristine A. Viscovich, ANP

***Indiana University* (Indianapolis, IN)**

David G. Marrero, PhD*

Melvin J. Prince, MD*

Susie M. Kelly, RN, CDE**

Yolanda F. Dotson, BS

Edwin S. Fineberg, MD

John C. Guare, PhD

Angela M. Hadden

James M. Ignaut, MA

Marcia L. Jackson

Marion S. Kirkman, MD

Kieren J. Mather, MD

Beverly D. Porter, MSN

Paris J. Roach, MD

Nancy D. Rowland, BS, MS

Madelyn L. Wheeler, RD

***Medstar Research Institute* (Washington, DC)**

Robert E. Ratner, MD*

Gretchen Youssef, RD, CDE**

Sue Shapiro, RN, BSN, CCRC**

Catherine Bavido-Arrage, MS, RD, LD

Geraldine Boggs, MSN, RN

Marjorie Bronsord, MS, RD, CDE

Ernestine Brown

Wayman W. Cheatham, MD

Susan Cola

Cindy Evans

Peggy Gibbs

Tracy Kellum, MS, RD, CDE

Claresa Levatan, MD

Asha K. Nair, BS

Maureen Passaro, MD

Gabriel Uwaifo, MD

***University of Southern California/UCLA Research Center* (Alhambra, CA)**

Mohammed F. Saad, MD*

Maria Budget**

Sujata Jinagouda, MD**

Khan Akbar, MD

Claudia Conzues

Perpetua Magpuri

Kathy Ngo

Amer Rassam, MD

Debra Waters

Kathy Xapthalamous

***Washington University* (St. Louis, MO)**

Julio V. Santiago, MD* (deceased)

Samuel Dagogo-Jack, MD, MSc, FRCP, FACP*

Neil H. White, MD, CDE*

Samia Das, MS, MBA, RD, LD**

Ana Santiago, RD**

Angela Brown, MD

Edwin Fisher, PhD

Emma Hurt, RN

Tracy Jones, RN

Michelle Kerr, RD

Lucy Ryder, RN

Cormarie Wernimont, MS, RD

***Johns Hopkins School of Medicine* (Baltimore, MD)**

Christopher D. Saudek, MD*

Vanessa Bradley, BA**

Emily Sullivan, MEd, RN**

Tracy Whittington, BS**

Caroline Abbas

Frederick L. Brancati, MD, MHS

Jeanne M. Clark, MD

Jeanne B. Charleston, RN, MSN

Janice Freel

Katherine Horak, RD

Dawn Jiggetts

Deloris Johnson

Hope Joseph

Kimberly Loman

Henry Mosley

Richard R. Rubin, PhD

Alafia Samuels, MD

Kerry J. Stewart, EdD

Paula Williamson

***University of New Mexico* (Albuquerque, NM)**

David S. Schade, MD*

Karwyn S. Adams, RN, MSN**

Carolyn Johannes, RN, CDE**

Leslie F. Atler, PhD

Patrick J. Boyle, MD

Mark R. Burge, MD

Janene L. Canady, RN, CDE

Lisa Chai, RN

Ysela Gonzales, RN, MSN

Doris A. Hernandez-McGinnis

Patricia Katz, LPN

Carolyn King

Amer Rassam, MD

Sofya Rubinchik, MD

Willette Senter, RD

Debra Waters, PhD

***Albert Einstein College of Medicine* (Bronx, NY)**

Harry Shamoon, MD*

Janet O. Brown, RN, MPH, MSN**

Elsie Adorno, BS

Liane Cox, MS, RD

Jill Crandall, MD

Helena Duffy, MS, C-ANP

Samuel Engel, MD

Allison Friedler, BS

Crystal J. Howard-Century, MA

Stacey Kloiber, RN

Nadege Longchamp, LPN

Helen Martinez, RN, MSN, FNP-C

Dorothy Pompi, BA

Jonathan Scheindlin, MD

Elissa Violino, RD, MS

Elizabeth Walker, RN, DNSc, CDE

Judith Wylie-Rosett, EdD, RD

Elise Zimmerman, RD, MS

Joel Zonszein, MD

***University of Pittsburgh* (Pittsburgh, PA)**

Trevor Orchard, MD*

Rena R. Wing, PhD*

Gaye Koenning, MS, RD**

M. Kaye Kramer, BSN, MPH**

Susan Barr, BS

Miriam Boraz

Lisa Clifford, BS

Rebecca Culyba, BS

Marlene Frazier

Ryan Gilligan, BS

Susan Harrier, MLT

Louann Harris, RN

Susan Jeffries, RN, MSN

Andrea Kriska, PhD

Qurashia Manjoo, MD

Monica Mullen, MHP, RD

Alicia Noel, BS

Amy Otto, PhD

Linda Semler, MS, RD

Cheryl F. Smith, PhD

Marie Smith, RN, BSN

Elizabeth Venditti, PhD

Valarie Weinzierl, BS

Katherine V. Williams, MD, MPH

Tara Wilson, BA

***University of Hawaii* (Honolulu, HI)**

Richard F. Arakaki, MD*

Renee W. Latimer, BSN, MPH**

Narleen K. Baker-Ladao, BS

Ralph Beddow, MD

Lorna Dias, AA

Jillian Inouye, RN, PhD

Marjorie K. Mau, MD

Kathy Mikami, BS, RD

Pharis Mohideen, MD

Sharon K. Odom, RD, MPH

Raynette U. Perry, AA

***Southwest American Indian Centers* (Phoenix, AZ; Shiprock, NM; Zuni, NM)**

William C. Knowler, MD, DrPH*^+^

Norman Cooeyate**

Mary A. Hoskin, RD, MS**

Carol A. Percy, RN, MS**

Kelly J. Acton, MD, MPH

Vickie L. Andre, RN, FNP

Rosalyn Barber

Shandiin Begay, MPH

Peter H. Bennett, MB, FRCP

Mary Beth Benson, RN, BSN

Evelyn C. Bird, RD, MPH

Brenda A. Broussard, RD, MPH, MBA, CDE

Marcella Chavez, RN, AS

Tara Dacawyma

Matthew S. Doughty, MD

Roberta Duncan, RD

Cyndy Edgerton, RD

Jacqueline M. Ghahate

Justin Glass, MD

Martia Glass, MD

Dorothy Gohdes, MD

Wendy Grant, MD

Robert L. Hanson, MD, MPH

Ellie Horse

Louise E. Ingraham, MS, RD, LN

Merry Jackson

Priscilla Jay

Roylen S. Kaskalla

David Kessler, MD

Kathleen M. Kobus, RNC-ANP

Jonathan Krakoff, MD

Catherine Manus, LPN

Sara Michaels, MD

Tina Morgan

Yolanda Nashboo (deceased)

Julie A. Nelson, RD

Steven Poirier, MD

Evette Polczynski, MD

Mike Reidy, MD

Jeanine Roumain, MD, MPH

Debra Rowse, MD

Sandra Sangster

Janet Sewenemewa

Darryl Tonemah, PhD

Charlton Wilson, MD

Michelle Yazzie

***George Washington University Biostatistics Center (DPP Coordinating Center* Rockville, MD)**

Raymond Bain, PhD*

Sarah Fowler, PhD*

Tina Brenneman**

Solome Abebe

Julie Bamdad, MS

Jackie Callaghan

Sharon L. Edelstein, ScM

Yuping Gao

Kristina L. Grimes

Nisha Grover

Lori Haffner, MS

Steve Jones

Tara L. Jones

Richard Katz, MD

John M. Lachin, ScD

Pamela Mucik

Robert Orlosky

James Rochon, PhD

Alla Sapozhnikova

Hanna Sherif, MS

Charlotte Stimpson

Marinella Temprosa, MS

Fredricka Walker-Murray

***Central Biochemistry Laboratory* (Seattle, WA)**

Santica Marcovina, PhD, ScD*

Greg Strylewicz, PhD**

F. Alan Aldrich

***Carotid Ultrasound***

Dan O’Leary, MD*

***CT Scan Reading Center***

Elizabeth Stamm, MD*

***Epidemiological Cardiology Research Center- Epicare* (Winston-Salem, NC)**

Pentti Rautaharju, MD, PhD*

Ronald J. Prineas, MD, PhD*/*

Teresa Alexander

Charles Campbell, MS

Sharon Hall

Yabing Li, MD

Margaret Mills

Nancy Pemberton, MS

Farida Rautaharju, PhD

Zhuming Zhang, MD

***Nutrition Coding Center* (Columbia, SC)**

Elizabeth Mayer-Davis, PhD*

Robert R. Moran, PhD**

***Quality of Well-Being Center* (La Jolla, CA)**

Ted Ganiats, MD*

Kristin David, MHP*

Andrew J. Sarkin, PhD*

***NIH/NIDDK* (Bethesda, MD)**

R. Eastman, MD

Judith Fradkin, MD

Sanford Garfield, PhD

***Centers for Disease Control & Prevention* (Atlanta, GA)**

Edward Gregg, PhD

Ping Zhang, PhD

***University of Michigan* (Ann Arbor, MI)**

William H. Herman, MD, MPH

## ^+^Genetics Working Group

Jose C. Florez, MD, PhD^1, 2^

David Altshuler, MD, PhD^1, 2^

Paul I.W. de Bakker, PhD^2^

Paul W. Franks, PhD, MPhil, MS^6, 7^

Robert L. Hanson, MD, MPH^3^

Kathleen Jablonski, PhD^5^

William C. Knowler, MD, DrPH^3^

Jarred B. McAteer, AB^1, 2^

Toni I. Pollin, PhD^4^

Alan R. Shuldiner, MD^4^

1=Massachusetts General Hospital

2=Broad Institute

3=NIDDK

4=University of Maryland

5=Coordinating Center

6=Lund University, Sweden

7=Harvard School of Public Health
